# The role and mechanisms of DNA methylation in the oocyte

**DOI:** 10.1042/EBC20190043

**Published:** 2019-11-29

**Authors:** Gintarė Sendžikaitė, Gavin Kelsey

**Affiliations:** 1 Epigenetics Programme, Babraham Institute, Cambridge CB22 3AT, U.K.; 2 Centre for Trophoblast Research, University of Cambridge, Cambridge CB2 3EG, U.K.

**Keywords:** chromatin, imprinting, methylation, oocytes

## Abstract

Epigenetic information in the mammalian oocyte has the potential to be transmitted to the next generation and influence gene expression; this occurs naturally in the case of imprinted genes. Therefore, it is important to understand how epigenetic information is patterned during oocyte development and growth. Here, we review the current state of knowledge of *de novo* DNA methylation mechanisms in the oocyte: how a distinctive gene-body methylation pattern is created, and the extent to which the DNA methylation machinery reads chromatin states. Recent epigenomic studies building on advances in ultra-low input chromatin profiling methods, coupled with genetic studies, have started to allow a detailed interrogation of the interplay between DNA methylation establishment and chromatin states; however, a full mechanistic description awaits.

## Introduction

All cells within an organism have the same genome but acquire different appearance and function. The identity of a cell is defined by selective activation of transcriptional programmes and subsequent maintenance during cell division. Epigenetic mechanisms, such as DNA methylation and histone tail post-translational modifications (PTMs), play a crucial role in cell lineage specification during development and faithful maintenance during cell division by regulating chromatin function [1].

### DNA methylation

DNA methylation is a covalent modification in which a methyl group from the donor S-adenosyl methionine (SAM) is attached to the carbon-5 atom of cytosine residues by DNA methyltransferases (DNMTs) [2,3]. In vertebrate genomes, it is found mostly, but not exclusively, within a CpG dinucleotide context. Since CG dyads are methylated symmetrically, i.e. on both DNA strands, their methylation can be heritable during cell division, thus providing the means for ‘epigenetic memory’ [4]. Although CpG dinucleotides are under-represented in vertebrate genomes, they are mostly methylated in somatic tissues. A notable exception is CpG islands (CGIs), where CpGs are clustered together [5,6]. Many CGIs found within gene promoters and transcription start sites (TSSs) are constitutively unmethylated, but some exhibit a lineage-specific DNA methylation status that helps shape the transcriptional landscape. Overall DNA methylation is considered to be a repressive mark, especially at heterochromatin, pericentromeric regions, gene promoters, repetitive and transposable elements [7]. In contrast, methylation over gene bodies is associated with active transcription. Functionally, DNA methylation alters binding of transcription factors and other chromatin interacting proteins, chromatin structure and accessibility, thus fine-tuning gene expression [7].

### Histones

In order to be packaged into the cell nucleus, DNA is wrapped around nucleosomes, octamers containing two each of histones H2A, H2B, H3 and H4. Histones have protruding terminal tails that can acquire a plethora of PTMs, such as methylation or acetylation [8]. In addition to PTMs, histones have non-canonical variants that are often incorporated outside DNA replication and which can add another layer of chromatin control. Many histone PTMs are associated with specific genomic regions, activity states and functions. For example, histone 3 lysine 4 trimethylation (H3K4me3) is found at active promoters and TSSs and is associated with gene activation [9], while H3K36me3 marks actively transcribed gene bodies and prevents spurious initiation at cryptic intragenic TSSs [10,11]. Similarly, developmental regulator genes can be marked by both activating H3K4me3 and repressive H3K27me3 marks, and are referred to as being ‘bivalent’ or ‘poised’ for transient activation [12]. Together, DNA methylation and histone PTMs control chromatin accessibility and packaging, allowing gene activation or repression.

### Epigenetic transitions in development

The ability of chromatin to undergo dynamic transitions is especially important during gamete and early embryo development, when two major epigenetic reprogramming waves are observed in mammals. Reprogramming is required to abolish established patterns determining cell lineage and to restore pluripotent potential [13–15]. Both sperm and egg are terminally differentiated gametes. After fertilisation, embryonic cells undergo epigenetic reprogramming to erase the gametic epigenome and regain totipotency [16]. During pre-implantation development, paternal DNA is rapidly demethylated, by a mechanism that is still not fully understood, partly involving activity of Ten-eleven translocation (TET) enzymes, while the maternal DNA methylation is lost in a passive manner during cellular proliferation. By the time the inner cell mass of the blastocyst is formed, the DNA methylation and histone PTM patterns that characterised the gametes are almost completely lost and only a small subset of gamete differentially methylated regions (gDMRs) and histone PTMs are retained [16,17]. After implantation, from the epiblast stage to gastrula (in the mouse between embryonic days E4.5 and E6.5), DNA methylation is regained and established in a lineage-specific pattern [18].

**Figure 1:**
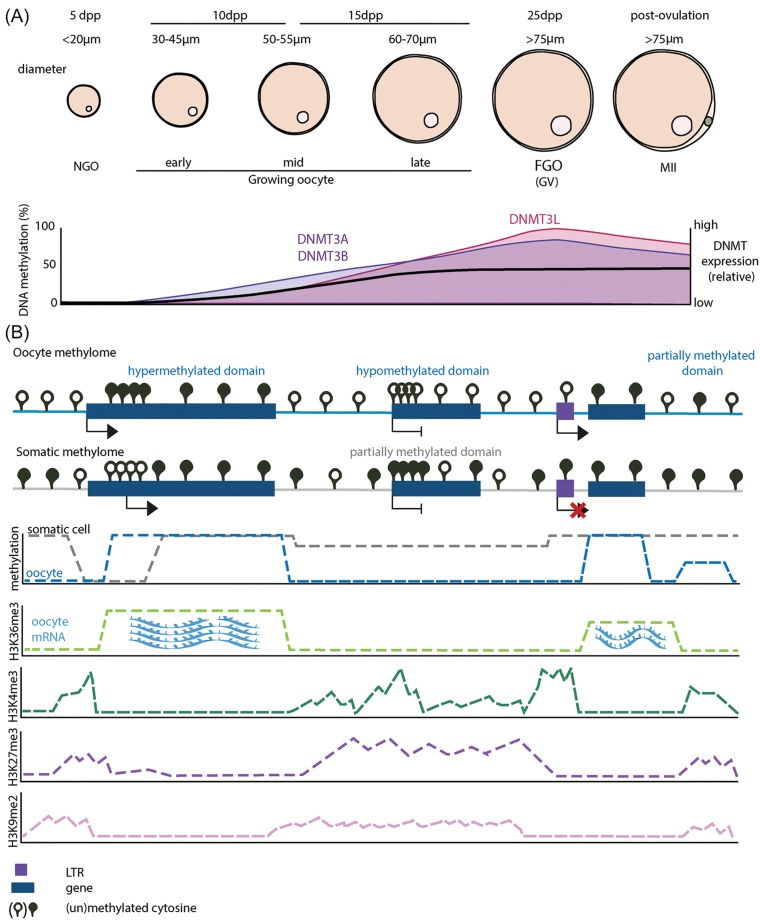
DNA methylation and histone mark patterns in oocyte (**A**) At day 5 post-partum (5dpp), the majority of oocytes are non-growing (NGOs). Oocytes gradually grow postnatally in mice, which is manifested by an increase in diameter. Representative sizes of oocyte at different dpps are shown. DNA metyltransferases DNMT3A, DNMT3B and DNMT3L are increasingly expressed from early growing oocytes, which coincides with the start of DNA methylation establishment. DNMT expression peaks in FGOs, when DNA methylation is completed at approximately 40% genome coverage. Ovulation initiates maturation of the FGO and transition to MII stage, when a polar body is formed (depicted in grey) and the MII oocyte remains arrested until fertilisation. Oocyte transcription is globally repressed at the MII stage, corresponding with decrease in DNMT3 transcript levels. DNA methylation is depicted by the black line, relative expression levels for DNMTA/DNMT3B and DNMT3L are in violet and magenta, respectively. Abbreviation: dpp, days post partum. (**B**) Schematic representation of DNA methylation and histone marks in the FGO. Unlike the methylome of somatic cells, which shows relatively high and even levels of DNA methylation across the genome, the oocyte methylome has distinct bimodal and clustered hyper- and hypo-methylation domains. In somatic cells, promoter CpG methylation status is linked to expression, where actively transcribed genes tend to have an unmethylated promoter, but in oocytes this relationship is more ambiguous. Hypermethylated domains in the oocyte are associated with actively transcribed genes and the H3K36me3 mark. Levels of H3K36me3 positively correlate with levels of gene expression at the locus. Notably, a subset of oocyte transcripts arise from oocyte-specific or LTR-driven promoters. Hypomethylated domains span transcriptionally inactive parts of the genome and only a small fraction of the oocyte methylome falls into a partially methylated domain category; these domains tend to overlap intergenic areas. H3K4me3 in the FGO has a non-canonical distribution, where it spreads from promoters, forming broad low to medium enrichment domains over hypo- and partially methylated domains, but not hypermethylated domains. Similarly, H3K27me3 has a non-canonical distribution over hypo- and partially methylated domains, some of which overlap H3K4me3, forming bivalent chromatin. Bivalent chromatin in the oocyte does not fully recapitulate bivalency found in embryonic tissues, as the enrichment of H3K27me3 is lower. Meanwhile, H3K9me2 covers approximately a quarter of oocyte genome, again, with exclusion of hypermethylated domains. Height of the curves indicates relative enrichment of histone marks. Abbreviation: LTR, long terminal repeat.

In mammals, the germline arises from somatic cells of the early post-implantation embryo. In mice, primordial germ cells (PGCs) are specified at E6.5–E7.25 in the yolk sac endoderm, and a second epigenetic reprogramming event is observed in these cells at E10.5–E11.5 [13,19]. During this time, parent-of-origin epigenetic marks are erased. As PGCs proliferate, they migrate to the genital ridge and differentiate into prospermatogonia or oogonia, depending on gonadal sex. Gamete-specific epigenomes are established in the germline soon after birth in male and in adulthood in female mice. Oogonia in the fetal ovary arrest at prophase I of the first meiotic division, and after puberty oocytes develop in readiness for ovulation and resumption of meiosis prior to fertilisation [20]. During this prolonged non-replicative period the oocyte develops from the primary non-growing oocyte (NGO) to the fully grown oocyte (FGO) germinal vesicle (GV) stage (Figure 1A), ultimately experiencing transcriptional arrest. With ovulation, the GV breaks down and the oocyte attains the meiosis II (MII) stage, where it remains until fertilisation. The process of oocyte development is accompanied by global transcriptional and epigenetic changes that are crucial for successful fertilisation and later development (Figure 1A) [21].

### Aim of this review

Although epigenetic reprogramming during early embryogenesis erases much of the gametic epigenomes, there are epigenetic features inherited from the oocyte and subsequently maintained in offspring. This is exemplified by imprinted genes, which are monoallelically expressed in offspring on account of DNA methylation differences acquired on these genes in oocytes compared with sperm [22]. This legacy of the gametic epigenome could provide the potential by which genetic and environmental factors that affect the oocyte epigenetic landscape give rise to intergenerational epigenetic inheritance [23], although the extent to which this occurs is still unclear. Nevertheless, it underlines the importance of understanding the normal processes of epigenetic programming that occur in the oocyte. In this respect, CpG methylation is of particular interest not only because the methylation pattern is remarkably different between male and female gametes, but also because of its potential for maintenance after cell division. Complete or partial loss of oocyte methylation is known to cause embryonic lethality and congenital diseases, highlighting the importance of faithful methylome establishment. This review focusses on established and recent knowledge of chromatin dynamics and key mechanisms responsible for faithful DNA methylation establishment in the oocyte. We discuss findings in mouse oocytes as a paradigm for mammalian systems.

## Oocyte DNA methylation and chromatin dynamics

### Oocyte methylation landscape

The oocyte is a terminally differentiated cell with a unique DNA methylation pattern, distinct from sperm or soma. In sperm DNA methylation is evenly dispersed and covers approximately 90% of the genome, with the notable exception of most CGIs that escape methylation. Meanwhile, mature oocytes show 40% global DNA methylation. Consequently, there are thousands of germline differentially methylated domains (gDMRs) [24–26].

The acquisition of DNA methylation in the oocyte is gradual throughout its growth and development. NGOs present in the primordial follicle before folliculogenesis is initiated are practically unmethylated, and methylation is acquired as the oocyte increases in size, primarily at the later stages of follicle development [24,25] (Figure 1A). What initiates *de novo* methylation is unclear, but might simply be the availability of appropriate DNMT activity coupled with permissive underlying chromatin state (see below). There does not seem to be much selectivity in the timing at which different genomic features become methylated, although there is a pronounced asynchrony amongst them, including CGIs and imprinted gDMRs [27–29]. Unlike somatic cells, where most CpGs are methylated, with the exception of active gene promoters, CpGs in FGOs exhibit a distinctly bimodal and clustered methylation distribution. Large genomic domains that are either hyper- (>75%) or hypo- (<25%) methylated form a signature oocyte methylome (Figure 1B). Only a small fraction of CpGs fall into a partially methylated category, and these domains are usually found at intergenic areas. This bimodal pattern is conserved in all mammalian oocytes so far studied [30,31].

Additionally, the oocyte has relatively high levels of non-CpG (CpH) methylation. It occurs mostly, but not exclusively, in the context of CpA dinucleotides [32,33]. CpG and CpH DNA methylation show very strong positive correlation and similar dynamics during oocyte maturation. CpH methylation is absent from NGOs and increases until and possibly beyond the FGO stage, where it is mostly found over active genes [25,29,33]. The significance of CpH methylation is unclear, but a new hypothesis has recently been advanced of its possible role in transcriptional regulation in human oocytes (https://www.biorxiv.org/content/10.1101/651141v1.full).

### The functional role of DNA methylation in the oocyte

DNA methylation appears to be dispensable for oocyte development and competence, as genetic ablation of oocyte methylation allows for successful fertilisation and ensuing embryonic development until the mid-gestation stage [34–38]. But it is essential for genomic imprinting: a subset of the gDMRs that evade embryonic reprogramming during early development result in parent-of-origin specific gene expression of the associated genes – imprinted genes. Failure to establish imprints leads to lethality or congenital diseases in both mice and humans, the pathologies observed are linked to placental and foetal growth, brain and metabolic function. Interestingly, only three imprinted loci are conferred by methylation in male gametes, while there are at least 26 DNA methylation-dependent imprinted regions conferred in the oocyte [22,24,25]. Imprinted loci contain a single or a cluster of genes, whose expression patterns are determined by imprinting control region (ICR) DNA methylation status. DNA methylation at ICRs is set up during oocyte growth in a transcription-dependent manner. For example, at the imprinted *Gnas* locus, transcriptional activity from an oocyte-specific promoter upstream of the *Nesp* gene is required for methylation of that locus. Disruption of transcription leads to failure of DNA methylation establishment over the ICR disrupting monoallelic expression of *Gnas* locus imprinted genes and viability in mice [39]. Oocyte ICRs are composed of CGIs enriched for a specific CG-rich hexameric motif recognised by the KRAB zinc-finger protein 57 (ZFP57). ZFP57 protects imprinted sites from demethylation during embryonic reprogramming by recruiting KAP1, SETDB1, HP1 and NP95 to form a robustly silenced locus [40,41]. Imprinted genes exhibit stable and heritable monoallelic parent-of-origin specific gene expression that can be maintained throughout the lifecourse, and which is only overwritten during PGC reprogramming. The complex mechanisms of ICR establishment and maintenance have been extensively studied and recently reviewed [22,42].

Apart from ICRs, some gDMRs show transient or tissue-specific inheritance post fertilisation [43,44]. Maternal non-imprinted gDMRs were shown to play a role in regulation of placental development in mice and humans [38,45]. The majority of such loci lose parent-of-origin methylation upon implantation, but it is currently not well understood how they affect pre-implantation development; whether gDMRs have any impact on zygotic genome activation, and if unaccounted demethylation escapees serve a more general purpose.

Complete loss of DNA methylation in oocytes manifests in embryonic lethality at E10.5, which was originally attributed to defects arising from imprint loss [34,36]. However, more recent evidence from various mouse knockouts (discussed in relevant contexts below) suggest that disruption of the oocyte methylome could lead to developmental defects unrelated to effects of disrupted imprinting (Table 1). For example, oocyte-ablation of *Kdm1a* or *Mll2*, which impair few or no imprints, respectively, show that minor global loss of gene-body methylation results in maternal-to-zygotic transcriptional transition or ovulation failure [46–49]. However, the effects of *Kdm1a* and *Mll2* knockouts could also be mediated by chromatin alterations. Meanwhile, *Stella* and *Uhrf1* knockout oocytes, which also show limited alteration to imprints but a strong global change in DNA methylation, arrest at the blastocyst stage [50–52]. Together, these findings suggest that although DNA methylation is not required for development or maturation of the oocyte and fertilisation *per se*, it is indispensable for embryonic development beyond imprinting in ways that are not yet fully understood.

**Table 1:** Summary of known genetic oocyte-specific knockout models and their impact on DNA methylation

Factor	Function	Impact on imprinted gDMRs in oocyte	Impact on global oocyte DNA methylation	Impact on post-fertilisation development	Reference
**Dnmt3a**	DNA methylation	Severe loss of DNA methylation	Global loss of DNA methylation	Incorrect imprint establishment, E9.5-E10.5 lethality	[24,33,34,35]
**Dnmt3b**	DNA methylation	Not affected	Not affected	Normal germ cell and post-fertilisation development	[33,34,35]
**Dnmt3l**	*De novo* methylation targeting	Severe loss of DNA methylation	Global loss of DNA methylation	Incorrect imprint establishment, E9.5-E10.5 lethality	[24,33,35,36]
**Dnmt1**	Maintenance of DNA methylation	Not affected	Slight loss of global DNA methylation, mostly at hemimethylated sites	Partial failure to maintain imprinted gDMRs, prenatal lethality	[79,80]
**Kdm1a**	H3K4me1/2 and H3K9me2 demethylase	Loss of methylation at *Gnas1A, Cdh15*	Minor loss of genic DNA methylation	Arrest at two-cell stage	[46,48,61]
**Kdm1b**	H3K4me1/2 demethylase	DNA methylation loss mostly at late methylating gDMRs	Loss of genic DNA methylation	E10.5 lethality	[46,60]
**Mll2**	H3K4me2/3 methyltransferase	Not affected	Minor loss of gene body methylation due to decreased transcription	Oocytes fail to ovulate and die prior to fertilisation	[47,49]
**Setd2**	H3K36me3 methyltrasnferase	Loss of methylation at all imprints	Global inverse pattern, hypermethylated domains lose methylation, hypomethylated domains gain methylation	Preimplantation lethality; post-implantation lethality in cytosolic rescue	[102,104]
**Uhrf1**	Recruitment of DNMT1 to hemimethylated DNA	Significant loss only at *Gnas1A, Peg10, Mest*	Minor loss of global DNA methylation, mostly over intermediately methylated and inactive domains	Lethality around blastocyst stage	[109]
**Stella**	Protection of genome from methylation	Not affected	Two-fold global hypermethylation	Lethality around blastocyst stage	[51,52]
**G9a**	H3K9me2 transferase, DNA methylation recruitment	Minor loss of methylation at *Gnas1A, Mest*	Minor loss of DNA methylation	Blastocyst or peri-implantation stage lethality (not fully penetrant)	[119,128]
**Sall4**	Transcription factor	Loss of methylation at all imprints	Major whole genome DNA methylation loss	Oocytes fail to mature	[120]
**Hdac1/Hdac2**	Histone deacetylases	Loss of methylation at all imprints	Global loss of DNA methylation	Oocytes fail to mature	[62,63]
**Sin3a**	Member of HDAC repressor complex	Loss of methylation at selected imprints	n/a	Lethality at two-cell stage (knockdown experiment)	[62,126,127]
**Cfp1**	SETD1 H3K4 methyltransferase DNA binding subunit	n/a	Global loss of DNA methylation	Lethality at two-cell stage	[89]
**Hira**	H3.3 deposition chaperone	Reduction in DNA methylation at imprints	Global loss of DNA methylation	Lethality immediately after fertilisation	[58]

### Transcription and transposable elements

With the recruitment of primordial follicles into growth (from NGO to early growing oocyte), a definitive oocyte transcriptome is established. Once development has progressed to about the antral follicle stage (early to mid growing oocyte), expressed genes in oocytes start to acquire DNA methylation across their gene bodies. Methylation increases during oocyte growth and is completed by the FGO GV stage. High transcriptional levels, DNA methylation and H3K36me3 abundance show high correlation, and approximately 90% of methylome establishment can be attributed to transcription events (Figure 1B) [24–26,29,46,53]. In agreement with this, loss of transcription upstream of imprinted genes results in failure to set up methylation at ICRs and imprinting of these genes [26,39,54]. In the oocyte, subsets of transposable elements, especially Long Terminal Repeats (LTRs), are very active and highly expressed: they can act as promoters, TSSs or splice donors, thereby generating approximately 10% of oocyte-specific transcript species [26,55,56]. Transcriptional activity of these LTRs contributes to the generation of hypermethylated domains found downstream (Figure 1B) [26]. A recent study in mouse, rat and human oocytes identified that approximately one-sixth of all DNA methylation is linked to transcription initiated at LTRs [31]. LTR-dependent DNA methylation shows strong species specificity, and can be inherited to blastocyst or extraembryonic tissues [31]. Moreover, LTRs are suggested to be drivers of species-specific imprint establishment in humans and mice (https://www.biorxiv.org/content/biorxiv/early/2019/08/07/723254.full.pdf).

### Local chromatin environment

As noted above, not all sites gain methylation simultaneously during oocyte growth [24,27,29]. The timing of DNA methylation of specific genes and genomic features is not linked to the underlying sequence but could rather be assigned to local chromatin environment, histone PTMs and nucleosome density. Although the oocyte is in a non-replicative state, nucleosome turnover, an inherent process during transcription, is required to aid oocyte maturation. Deletion of HIRA, a histone chaperone responsible for non-canonical histone deposition in quiescent cells [57], in the oocyte results in increased accessibility and loss of landmark histone modifications, which in turn leads to genome-wide hypomethylation [58]. At the same time, nucleosome depletion at certain sites increases accessibility and could allow easier access for DNMTs. Genes showing high accessibility at TSSs or across the gene body during oocyte development are associated with higher transcription and DNA methylation levels [53]. Genes that remain highly compacted throughout oocyte growth tend to remain silent and are not subjected to *de novo* methylation. Similarly, precocious expression of the *de novo* methyltransferases DNMT3A and DNMT3L accelerates imprint establishment at only a selection of loci and others appear to be protected by a restrictive chromatin environment [59].

Loci that acquire methylation late in oocyte growth are often CGI-rich, and require removal of H3K4me2 or H3K4me3, active chromatin marks that inhibit binding and activity of the DNMT3A/L complex [29,46]. The H3K4 demethylases KDM1A and KDM1B are expressed throughout oocyte growth or from mid-growth phase, respectively. Ablation of KDM1B, and to some extent KDM1A, in the oocyte resulted in failure to establish full DNA methylation over most imprinted genes and led to focal hypomethylation [46,48,60,61]. Similarly, histone deacetylase 1 and 2 (HDAC1/2) are expressed in early oocytes, with the former subsequently decreasing as growth progresses. Loss of HDAC1/2 results in altered chromatin environment and perturbed transcription, leading to both global and imprint-specific DNA methylation loss in the oocyte [62,63]. As noted above, 10% of DNA methylation in the oocyte is transcription-independent and these loci tend to be methylated quite late in oocyte growth. DNMT targeting to those sites is likely to involve histone modifications, remodellers or other chromatin-interacting proteins that would make local chromatin accessible and appropriately marked, but the precise mechanism(s) are unknown.

### DNA methylation machinery

Although the oocyte has a unique DNA methylation pattern, it relies on an otherwise conventional DNA methylation machinery. The DNMT family in mammals consists of five members: one maintenance, three *de novo* methyltransferases and a cofactor. DNMT1, the maintenance DNMT, recognises and methylates the unmethylated strand on hemimethylated DNA [64]. During S phase, DNMT1 associates with replication foci through Ubiquitin-like, plant-homoeodomain (PHD) and ring finger-containing 1 (UHRF1) and ensures faithful methylation maintenance on the nascent DNA strand [65]. Homozygous deletion of *Dnmt1* results in embryonic lethality [66]. In addition to DNMT1, there are three *de novo* DNMTs, which use unmethylated DNA as a substrate. DNMT3A and DNMT3B show partial redundancy and are both required for epigenetic reprogramming during embryogenesis [67]. *Dnmt3a^−/−^* mice fail to survive longer than 3 weeks postnatally, while *Dnmt3b^−/−^* and *Dnmt3a^−/−^ Dnmt3b^−/−^* mice die before E11.5. DNMT3C, a third, murine-specific *de novo* methyltransferase, has recently been discovered; it silences evolutionary young retrotransposons in prospermatogonia by methylating their promoters [68]. DNMT3L is the odd member of DNMT family, since it does not have an active catalytic domain [69]. DNMT3L is also less conserved between the species and is only found in mammals with genomic imprinting [70]. The C-terminal domain of DNMT3L can bind DNMT3A and DNMT3B C-terminal domains and significantly enhances their chromatin binding and/or catalytic activity by formation of tetramers [71,72].

#### DNMTs in the oocyte

Both DNMT3A and DNMT3B are detectable and localise to the nucleus of the GV oocyte [73]. However, DNMT3A and DNMT3L are the key players, both necessary for faithful DNA methylation establishment. Expression of DNMT3A, DNMT3B and DNMT3L in growing oocytes is coordinated, their expression levels increase as oocyte development proceeds, peaking towards the GV stage when *de novo* methylation is complete, and decrease once oocyte attains the MII stage (Figure 1A) [74]. DNMT3A is essential for catalysing the methylation, but relies heavily on interaction with DNMT3L for genomic targeting [24,25,34–36]. Both *Dnmt3a^−/*−*^* or *Dnmt3l^−/−^* mice fail to establish germline methylation, but *Dnmt3l^−/−^* show a more severe phenotype [24,34–36,72,75]. In mice, the conditional deletion of DNMT3A or DNMT3L results in failure to establish DNA methylation in the oocyte and consequently a loss of maternal imprints in offspring [34–36]. DNMT3B, although expressed, does not appear to play a role in DNA methylation in the oocyte, and *Dnmt3b^−/−^* oocytes mature without failure and have unaffected phenotype [35]. However, in other contexts [76], DNMT3B is able to bind DNMT3L. It is possible that in the *Dnmt3a^−/−^* background, DNMT3B interacts with DNMT3L to rescue some of the methylation targets. Unlikely to be required as a maternal transcript prior to zygotic genome activation, the role of *Dnmt3b* expression in the oocyte remains elusive. Regarding DNMT1, the oocyte expresses an oocyte-specific isoform *Dnmt1o*, which arises from an alternative 5′ exon and is 118 amino acids shorter than the somatic isoform. DNMT1O is found in high abundance in growing oocytes and, although some nuclear localisation is retained, it is mostly cytoplasmic [73,77]. DNMT1O in the oocyte has a minor role in fully methylating hemimethylated sites, but the main purpose for accumulation of this protein in oocytes is likely to be after fertilisation [33,78,79].

### Recruitment of DNMTs in the genomic context

**Figure 2:**
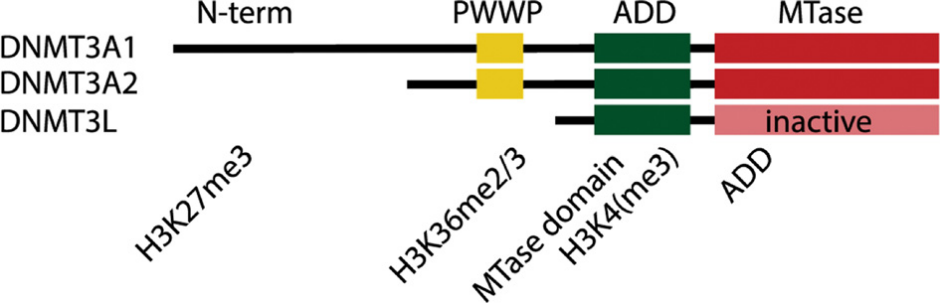
Schematic structures of DNMT3A and DNMT3L, and their predicted interactors N-terminal domain of DNMT3A1 is required for localisation at bivalent chromatin shores in ES cells. Notably, this isoform is not expressed in the oocyte. The PWWP domain is poised to recognise H3K36me2/3, but this interaction has not been interrogated in the oocyte. The ADD domain forms a fold with the catalytic MTase domain to create an inactive allosteric conformation of DNMT3A. Recognition of unmethylated H3K4 tail alters this conformation and stimulates catalytic activity, while H3K4me3 repels the protein.

Many studies have sought to understand how the oocyte-specific DNA methylation pattern is set. Overall, DNMT3s show rather limited target sequence specificity, which is also true for methylated CGIs in the oocyte [24,80]. However, N-terminal regulatory domains of these proteins – the ADD (ATRX-DNMT3-DNMT3L) and PWWP (Pro-Trp-Trp-Pro motif) domains – can interact with various histone PTMs and guide DNMT localisation and enzymatic activity (Figure 2). Recent advances in low-input chromatin immunoprecipitation methods, requiring as little as a few hundred cells [16], have allowed the interrogation of the localisation of histone marks with respect to specific genomic features and DNA methylation status. In combination with knockouts for specific histone modifier enzymes, such studies are shedding light on instructive and consequential interactions between different mechanisms. Since DNMT3A is responsible for the majority of DNA methylation in the oocyte, we focus on known and predicted mechanisms of its targeting to the genome.

#### The ADD domain, H3 and H3K4me3

The ADD domain, present in all DNMT3s, is homologous to a conserved PHD zinc finger motif. ADD domains of DNMT3A and DNMT3L have a high affinity to the N-terminal region of histone 3, especially when unmethylated at lysine 4 (H3K4). This interaction promotes DNA methylation catalysis [81–84]. When DNMT3A is in complex with DNMT3L, which is presumably the case in the oocyte, recruitment of the DNMT3L ADD domain is sufficient to engage the whole complex [72,81]. Methylated lysine H3K4me3 in somatic cells is found at active gene promoters and TSSs, and inhibits DNMT3A activity [81–84]. Structural studies have found that DNMT3A is intrinsically in an autoinhibitory allosteric conformation, driven by the ADD domain: the ADD domain masks the DNA binding site of the catalytic domain. Recognition of unmethylated H3K4 specifically allows a structural shift and uncouples the ADD-catalytic domain interaction, allowing activation of DNMT3A enzymatic function [85]. Thus, DNA methylation and H3K4me3 are mutually exclusive in the genome. Engineering of the ADD domain to lose sensitivity to H3K4me3 results in aberrant gain of methylation over these domains [86]. The function of the ADD domain in somatic cells is relatively well studied with many mechanistic insights supported by structural work. Current evidence of methylation patterns in the oocyte suggests that the ADD domain plays a similar role in the oocyte, although no oocyte-specific studies have been conducted.

Curiously, H3K4me3 has an atypical broad domain pattern in the mouse FGO, where it covers approximately one-fifth of the genome. Broad domains are found not only over TSSs but over distal regions as well, and these domains tend to show low or intermediate levels of DNA methylation (Figure 1B) [47,87,88]. H3K4me3 appears as canonical sharp peaks in NGOs, and these loci are protected from *de novo* DNA methylation through ADD domain inhibitory mechanism [81,82]. During oocyte development, H3K4me3 spreads, simultaneously but at mutually exclusive locations with DNA methylation, and invades intermediately methylated domains from the mid to late oocyte growth stage, reaching the final distribution in FGO [47,87,88]. In the oocyte, the H3K4me3 methyltransferase MLL2 (KMT2B), expressed at mid- to MII stages, is responsible for non-canonical H3K4me3 establishment [47,49]. In MLL2 knockout oocytes DNA methylation spreads to some but a limited number of domains that should normally contain H3K4me3, whereas in DNMT3A knockout oocytes there is a more pervasive spread of H3K4me3 into normally DNA methylated domains [47]. This observation suggests that DNMT3A recruitment depends not only on absence of antagonistic H3K4me3, but also presence of an attractive histone PTM. Another H3K4 methyltransferase, SETD1, is assumed to be responsible for canonical promoter-associated H3K4me3 establishment via its DNA binding subunit CFP1, although this has not yet been validated by ChIP-seq analysis. Deletion of CFP1 in the oocyte resulted in loss of global DNA methylation, however, this could be an indirect effect of DNMT3A down-regulation following loss of promoter H3K4me3 [89]. These findings suggest a complex interaction, where localised H3K4me3 at TSSs prevents DNA methylation establishment, while hypermethylation at transcription-independent domains protects these loci from broad non-canonical H3K4me3.

#### The PWWP domain, H3K36me3 and bivalent chromatin

The PWWP domain is a member of the Tudor domain royal superfamily and is mostly found in chromatin-interacting proteins. It has an intrinsic and somewhat unspecific affinity to chromatin and modified histones [90]. In the DNMT family, the PWWP domain is only found in DNMT3A and DNMT3B, and is known to be required for methylation of major satellite repeats [90–92]. The PWWP domain contains a conserved aromatic cage that enables binding of methylated lysines, especially H3K36me3 [93,94]. Extensive biochemical work suggests that the PWWP domain of DNMT3A interacts specifically and exclusively with H3K36me2/3 [95–98]. Dhayalan et al. [95] show that a mutation (D329A) within the aromatic cage of the DNMT3A PWWP domain disrupts binding of H3K36me3 *in vitro*. H3K36me3 is universally found over expressed gene bodies, and studies suggest that in mouse embryonic stem cells DNMT3B and not DNMT3A is responsible for DNA methylation over H3K36me3 domains [11,99], a conclusion supported by a study expressing DNMT3B in yeast [100]. We used a mouse model to investigate the effect of the DNMT3A^D329A^ mutation but did not find any evidence of methylation defects over gene bodies and H3K36me3 domains in embryos or adult mouse brain [101], or in oocytes (unpublished). This raised a question whether DNMT3A in the oocyte indeed was recruited by the transcription-dependent H3K36me3 mark over gene bodies [46].

Xu et al. [102] tested the DNA methylation and H3K36me3 interaction from a different perspective and generated a mouse with an oocyte-specific SETD2 knockout: SETD2 is the histone lysine methyltransferase uniquely responsible for H3K36me3 deposition in mammalian cells [103,104]. H3K36me3 is already present in early oocyte growth stages and increases together with transcription and DNA methylation, persisting until the MII stage (Figure 1B). Depletion of H3K36me3 in NGO resulted in dramatic loss and redistribution of DNA methylation and affected all maternal imprints in FGO. It also led to altered transcriptome, reorganisation of H3K4me3 and H3K27me3 mark landscapes, and caused embryonic lethality [102]. This work suggests that H3K36me3 is a master regulator of the oocyte methylome, and is required to prevent H3K4me3 and H3K27me3 from the spreading into actively transcribed regions. Yet it remains unclear whether the recognition of this histone tail modification by the DNMT3A PWWP domain is the main driver of DNA methylation establishment.

#### The N-terminal domain and H3K27me3

DNMT3A has two major isoforms. DNMT3A1, the longer isoform that predominates in adult somatic tissues, and DNMT3A2, a shorter isoform found in the oocyte and embryonic tissues. DNMT3A1 has been shown to localise preferentially to the shores of Polycomb-regulated bivalent chromatin and follows the dynamics of the H3K27me3 mark during neuronal differentiation [105]. Bivalent chromatin comprises an active chromatin mark H3K4me3 and repressive H3K27me3 and is protected from DNA methylation in somatic cells [12,106]. Bivalent domains are also present in the oocyte [47], although they appear to be less pronounced than in embryonic tissues (Figure 1B), while H3K27me3 shows a somewhat non-canonical distribution: it is anti-correlated with transcribed genes and mostly overlaps hypo- and partially methylated domains (Figure 1B) [47,107]. In soma, the DNMT3A and bivalent chromatin interaction depends on the N-terminal disordered domain that is specific to isoform 1. However, only DNMT3A2, lacking the N-terminal domain, is expressed in the oocyte [108]. Currently the mechanism of mutual exclusivity between DNA methylation and H3K27me3 at CGIs is not known. It is possible that lack of DNMT recruitment to bivalent chromatin shores simply results in those domains remaining unmethylated. This theory has not been put to test and H3K27me3 link to DNA methylation in the oocyte is unclear.

#### UHRF1, STELLA and DNMT1

While the loss of imprinted gDMRs in the oocyte causes mid-gestational embryonic lethality, excessive gain of methylation, even when gDMRs remain relatively unaffected, also results in failure of embryonic development [51,52], highlighting the importance of DNA methylation and lack thereof outside of the gene body context.

The UHRF1 ubiquitin ligase recognises hemimethylated DNA and recruits the maintenance DNMT1 to these sites [50,65]. UHRF1 is mostly cytoplasmic in the oocyte, however, when deleted, it results in loss of DNMT1 localisation to the nucleus [109]. UHRF1 knockout oocytes exhibit a decrease in global DNA methylation of approximately 8%, which is greater than what could be attributed to loss of symmetric 5mC at hemimethylated sites that is dependent on DNMT1, suggesting additional pathways of UHRF1-dependent methylation in oocytes. Interestingly the loss was observed in non-CpG methylation too, suggesting that UHRF1 is involved in *de novo* methylation targeting [109].

Work in NIH3T3 cells showed that UHRF1 is regulated by a maternal effect protein STELLA (also known as PGC7 and DPP3A). Overexpression of STELLA prevented localisation of UHRF1 and DNMT1 to the DNA replication fork and resulted in global hypomethylation [110]. STELLA is expressed in the oocyte and is known to be responsible for protection of 5mC during epigenetic reprogramming in early embryogenesis [111,112]. However, earlier work did not find any effect of *Stella* knockout in the oocyte [112]. Recently, two studies showed that knocking-out STELLA in the oocyte resulted in aberrant gain of DNA methylation of more than 28% [51,52]. In the context of the oocyte, STELLA is responsible for nuclear export of UHRF1 to the cytoplasm, thereby preventing methylation of regions normally unmethylated in the oocyte.

UHRF1 or STELLA knockouts in the oocyte cause hypo- and hyper-methylation, respectively, but the effect was localised to intermediately methylated regions harbouring low- or non-expressed genes [51,109]. Both proteins are required for proper embryonic development, and these knockouts result in early lethality. This work sheds some light on cellular regulation of DNMT1 in the oocyte, and highlights our lack of understanding of the roles of the UHRF1–DNMT1 interaction in *de novo* methylation. Notably, *Uhrf1* or *Stella* knockout oocytes exhibit severe global methylation changes that do not alter imprinting regions, yet embryos fail early in development [51,52,109], suggesting that oocyte methylation at non-imprinted domains has important consequences post-fertilisation.

#### G9A/GLP and H3K9me2

G9A/GLP (EHMT2/EHMT1) is a histone methyltransferase complex responsible for H3K9me2 primarily in euchromatic regions of the genome [113]. In somatic cells H3K9me2 is highly abundant and is involved in heterochromatin formation [114]. G9A initiates heterochromatinisation of the genome by establishing H3K9me2 during embryogenesis and, independent of its catalytic activity, recruits *de novo* DNMT3s for DNA methylation [115–117]. G9A is expressed from early oocyte stages, and levels of both G9A expression and H3K9me2 abundance increase as the oocyte progresses through the growth phase [118]. Broad domains of H3K9me2 cover more than a quarter of the mature oocyte genome but mostly where CpG methylation is low (Figure 1b) [119]. Oocyte-specific G9A knockout shows no effect on NGOs and only a slight loss of DNA methylation is observed in FGOs. Currently there are no studies conducted in the oocyte that explore GLP function, but the outcome is expected to be similar as in most situations G9A function is completely dependent on GLP. Thus, although H3K9me2 is abundant in the oocyte, it does not direct DNA methylation to specific genomic loci.

#### SALL4

In addition to chromatin modifications and modifiers, other DNA-interacting proteins, such as transcription factors, could be involved in DNA methylation regulation. The transcription factor SALL4 is expressed from the primary follicle stage throughout the growth of the oocyte, and is found in the nucleus. After the GV to MII transition, it relocates to the cytoplasm. Oocyte-specific ablation of *Sall4* shows that oocytes failed to reach the mature stage or undergo GV breakdown, required to proceed to the MII stage. Interestingly, *Sall4* knockout oocytes show loss of DNMT3A nuclear localisation and dramatically reduced levels of 5-mC. Imprinted regions were almost completely unmethylated, while repetitive elements were fairly hypomethylated. SALL4 is a transcriptional regulator of several histone lysine demethylases: *Sall4* knockout oocytes show higher expression of *Kdm5b*, and lower expression of *Kdm6a* and *Kdm6b*, consistent with lower H3Kme3 and higher H3K27me3 levels, respectively, as assessed by immunofluorescence [120]. This finding again links to the importance of dynamic chromatin changes in oocyte growth, and exemplifies the upstream involvement of transcription factors in DNA methylation.

## Summary and gaps in current knowledge

In summary, oocyte DNA methylation is established *de novo* by the DNMT3A/L complex after complete erasure during PGC specification. The methylation machinery is controlled at two levels: subcellular localisation limits availability, while interactions with chromatin environment cues guide catalytic activity. The majority of DNA methylation is associated with actively transcribed units and the transcription-associated histone mark H3K36me3, the remainder is likely dependent on other histone modifications that create permissive or antagonistic chromatin states. Recent insights, especially on the roles of UHRF1-STELLA-DNMT1, G9A, SALL4, MLL2 and of DNMT3A targeting domains, build on extensive DNA methylome, transcriptome and chromatin landscape maps to draw a bigger picture.

It is well understood that one of the major roles of the oocyte is accumulation of transcripts required prior to zygotic genome activation; establishment of imprinting is also of vital importance. Nonetheless, knockout models for a number of chromatin regulators clearly indicate that fidelity of the oocyte methylome has a significant impact on pre- and post-fertilisation embryonic development beyond imprinting (Table 1). The oocyte represents a unique non-replicative cellular environment where *de novo* DNA methylation is established on an essentially unmethylated background by a defined enzymatic machinery. These properties, as well as the biological importance in the potential for transgenerational inheritance, make it an interesting system to study fundamental laws of and exceptions to epigenetic mechanisms. The role of such defined hyper- and hypo-methylated domains, in comparison with the relatively hypermethylated genomes of somatic cells, is unclear. Failure to erase global hypermethylation inherited from *Stella* knockout oocytes in embryonic reprogramming suggests a limited capacity in the oocyte for reprogramming. Although DNA methylation and histone PTMs show strong associations, it is rather difficult to decipher the order or hierarchy of these in genomic localisation, as illustrated in the MLL2 study [47]. To add to the complexity, the oocyte histone landscape requires active maintenance and dynamic changes during growth and maturation [46,58,60,102], even in the absence of DNA replication.

It should be noted that the findings presented here are based mainly on *in vivo* work carried out in mouse. Difficulties in acquiring material and ethical considerations limit research possibilities in humans and create challenges for translation of this research [121]. Broadly, DNA methylation patterns and reprogramming after fertilisation are conserved between humans and mice [122,123]. Work by Brind’Amour et al. [31] shows that gene body methylation in the oocyte is shared amongst human, rat and mouse, but also reveal LTR-driven variability at intergenic regions. Recently, Xia et al. [124] reported the first study of histone PTM landscapes in human oocytes. Surprisingly, they did not find non-canonical distributions of H3K4me3 and H3K27me3, as observed in mice, and report a new priming H3K4me3 rearrangement occurring post fertilisation and prior to zygotic genome activation. Moreover, human oocytes also lack DNMT3L [125], an essential *de novo* methylation factor in mice, suggesting that enzymatic targeting could be different, but currently no mechanistic studies have been done. Technological advances have provided a much more detailed understanding of the germ cell methylome, but further research is required to dissect mechanistic order and consequences of the highly unique oocyte methylome in mouse and humans.

## Summary

- The oocyte has a unique methylome of hyper- and hypo-methylated domains that are gradually established by DNMT3A and DNMT3L during oocyte growth.
- The majority of methylated domains are associated with active transcription units, the remainder require local chromatin reorganisation.
- DNMT3A and DNMT3L are recruited to chromatin through their regulatory domain interactions with modified histone tails.
- Access of the DNA methylation machinery to the genome is regulated by cellular localisation of DNMTs and local chromatin environment.
- Although much of the oocyte methylome may be dispensable, faithful methylation establishment at both imprinted and non-imprinted loci is essential for embryonic development. Further work is needed to elucidate the role of the oocyte methylome in early embryogenesis and beyond.

## Abbreviations

ADD: ATRX-DNMT3-DNMT3L
CFP1: CxxC finger protein 1
CGI: CpG island
ChIP-seq: chromatin immunoprecipitation sequencing
DNMT: DNA methyltransferase
dpp: days post-partum
EHMT1/2: euchromatic histone lysine methyltransferase 1 and 2, same as GLP and G9A
FGO: fully grown oocyte
gDMR: gamete differentially methylated region
GLP: G9A-like protein, same as EHMT1
GV: germinal vesicle
G9A: same as EHMT2
HDAC1/2: histone deacetylase 1 and 2
HIRA: histone cell cycle regulator
HP1: heterochromatin protein 1
H2/3a/3b/4: histone 2 or 3a or 3b or 4
H3K4me1/2/3: histone 3 lysine 4 mono- or di- or trimethylation
H3K9me2: histone 3 lysine 9 dimethylation
H3K27me3: histone3 lysine 27 trimethylation
H3K36me2/3: histone 3 lysine 36 di- or trimethylation
ICR: imprinting control region
KAP1: KRAB-associated protein 1
KDM1A/B: lysine demethylase 1a or 1b
KRAB: Krüppel associated box
LTR: long terminal repeat
MLL2: myeloid/lymphoid or mixed-lineage leukaemia
MTase: methyltransferase domain
MII: meiosis II
NGO: non-growing oocyte
NP95: nuclear protein 95
PGC: primordial germ cell
PHD: plant-homoeodomain
PTM: post-translational modification
PWWP: Pro-Trp-Trp-Pro motif
SALL4: Sal-like protein 4
SAM: S-Adenosyl methionine
SETD1/2: histone-lysine N-methyltransferase
SETDB1: SET domain bifurcated histone lysine methyltransferase 1
SIN3A: SIN3 transcription regulator family member a
STELLA: also known as primordial germ cell protein 7 or developmental pluripotency-associated 3
TET: ten-eleven translocation
TSS: transcription start site
UHRF1: Ubiquitin-like, PHD and ring finger-containing 1
ZFP57: KRAB zinc-finger protein 57
5mC: 5-methylcytosine
